# Identification of a unique conserved region from a kinetoplastid genome of *Leishmania orientalis* (formerly named *Leishmania siamensis*) strain PCM2 in Thailand

**DOI:** 10.1038/s41598-023-46638-3

**Published:** 2023-11-10

**Authors:** Pornchai Anuntasomboon, Suradej Siripattanapipong, Sasimanas Unajak, Kiattawee Choowongkomon, Richard Burchmore, Saovanee Leelayoova, Mathirut Mungthin, Teerasak E-kobon

**Affiliations:** 1 Department of Genetics, Faculty of Science, Kasetsart University, Bangkok, Thailand; 2 Omics Center for Agriculture, Bioresources, Food, and Health, Kasetsart University (OmiKU), Bangkok, Thailand; 3 Department of Microbiology, Faculty of Science, Mahidol University, Bangkok, Thailand; 4 Department of Biochemistry, Faculty of Science, Kasetsart University, Bangkok, Thailand; 5 Glasgow Polyomics, College of Medical, Veterinary and Life Sciences, University of Glasgow, Glasgow, UK; 6 Department of Parasitology, Phramongkutklao College of Medicine, Bangkok, Thailand

**Keywords:** Computational biology and bioinformatics, Genetics

## Abstract

Mitochondrial DNAs (mtDNAs) appear in almost all eukaryotic species and are useful molecular markers for phylogenetic studies and species identification. Kinetoplast DNAs (kDNAs) are structurally complex circular mtDNA networks in kinetoplastids, divided into maxicircles and minicircles. Despite several kDNAs of many *Leishmania* species being examined, the kDNAs of the new species, *Leishmania orientalis* (formerly named *Leishmania siamensis*) strain PCM2, have not been explored. This study aimed to investigate the maxicircle and minicircle DNAs of *L. orientalis* strain PCM2 using hybrid genome sequencing technologies and bioinformatic analyses. The kDNA sequences were isolated and assembled using the SPAdes hybrid assembler from the Illumina short-read and PacBio long-read data. Circular contigs of the maxicircle and minicircle DNAs were reconstructed and confirmed by BLASTn and rKOMICs programs. The kDNA genome was annotated by BLASTn before the genome comparison and phylogenetic analysis by progressiveMauve, MAFFT, and MEGA programs. The maxicircle of *L. orientalis* strain PCM2 (18,215 bp) showed 99.92% similarity and gene arrangement to *Leishmania enriettii* strain LEM3045 maxicircle with variation in the *12s rRNA* gene and divergent region. Phylogenetics of the whole sequence, coding regions, divergent regions, and *12s rRNA* gene also confirmed this relationship and subgenera separation. The identified 105 classes of minicircles (402–1177 bp) were clustered monophyletically and related to the *Leishmania donovani* minicircles. The kinetoplast maxicircle and minicircle DNAs of *L. orientalis* strain PCM2 contained a unique conserved region potentially useful for specific diagnosis of *L. orientalis* and further exploration of this parasite population genetics in Thailand and related regions.

## Introduction

The genus *Leishmania* is a member of the order Trypanosomatidae and one of the most important infectious parasitic groups^1,2^. One characteristic of the Trypanosomatids is the presence of a vast DNA network known as kinetoplast DNAs (kDNAs) within the mitochondrion. This DNA network contains dozens of relaxed maxicircles (20–40 kb) encoded for mitochondrial rRNAs and protein subunits of the electron transport complexes, similar to other eukaryotes, and thousands of minicircles (0.5–2 kb) per species which encode most of the guide RNAs (gRNAs) involved in RNA editing (insertion or deletion of uridine) of some transcripts from the maxicircles^3–7^. The RNA editing process of *Leishmania tarentolae* has been widely studied as the best model among several trypanosomatids^8–10^. A single mitochondrion of *L. tarentolae* has approximately 5000–10,000 minicircles and 20–50 maxicircles^11^. The *L. tarentolae* maxicircle contains genes encoding for two small mitochondrial rRNAs (9S and 12S rRNAs), several proteins responsible for the electron transport chain, including cytochrome b (Cyb), cytochrome oxidase subunits I, II, and III (COI, COII, and COIII), NADH dehydrogenase subunits 1, 2, 3, 4, 5, 7, 8, and 9 (ND subunits), a ribosomal protein (RpS12), and four uncharacterized genes i.e., MURF2, MURF5 or uS3m (a component of the mitoribosome), G3 and G4 G-rich genes (components of the respiratory complex I)^12–14^. Some genes of the trypanosomes (*COIII*, *Cytosine-rich Region 4* (*CR4*), *ND9*, and *RpS12*) were dual-coded, resulting in different proteins^15–19^. The transcripts derived from the leishmanial genes required editing, i.e., the high- or pan-editing of *ND8*, *ND9*, *G3*, *G4*, *ND3*, and *RpS12* genes before the mRNA translation^20–22^. The large divergent region (DR) of the *L. tarentolae* maxicircle (approximately 12 kb in length) consists of numerous tandem repeats and is hypothesized to be a functionally unknown noncoding region^7,8^. Interestingly, another study in *Leishmania major* displayed different variations in the DR region compared between the promastigote and amastigote stages^23^.

Several previous studies uncovered the maxicircle DNA fragments from different *Leishmania* species. These findings included the discovery of the 8.4-kb maxicircle fragment from *L. major* strain MHOM/SU/73/5ASKH in 2008^24^, an extensive RNA editing of genes encoded from the maxicircle of *Leishmania donovani* in 2009^25^, and the identification of the 6.5-kb and 4.3-kb maxicircle fragments from *Leishmania braziliensis* strain MHOM/BR/75/M2904 in 2011^26^. Advancement of next-generation sequencing technologies allowed quick and high-throughput determination of the nuclear genomic sequences of several *Leishmania* species and strains^27–30^. Only a few mitochondrial genomes were characterized in *Leishmania*^31–33^. In 2019, Camocho et al. used a BLAST search to extract sequence contigs specific to the maxicircles and employed a circularity test to confirm the minicircles of three *Leishmania* species (*L. major*, *Leishmania infantum*, and *L. braziliensis*) from the genomic sequence contigs^31^. Comparison of the maxicircles from these three species showed a conserved structure and gene order, while the number of minicircle classes varied: 97 classes for *L. major*, 49 classes for *L. infantum*, and three classes for *L. braziliensis*. Most identified minicircles shared conserved sequence blocks called CSBs. Another study identified 114 minicircle classes from *L. tarentolae* strain LEM125 and 24 classes from the UC strain using the PacBio sequencing platform^13^. A comparative genome study of seven *L. infantum* isolates in 2020 showed intra-specific conservation of the maxicircle and 59 distinct minicircle classes^33^. These authors found that drug-resistant strains had unique minicircle class patterns and tended to harbour an increased copy number of certain minicircle classes. Structural variation within the maxicircle of *Leishmania panamensis* strains was also observed, for example, within the *ND5* and *rpl12* genes, particularly in the DR region^32^. These genetic variations in the maxicircle and minicircle DNAs would benefit phylogenetic interpretation and understanding of the virulence and resistance of *Leishmania* species and strains.

*Leishmania orientalis*, formerly named *Leishmania siamensis*, is a new *Leishmania* species associated with cutaneous and visceral leishmaniasis observed in immunocompromised and immunocompetent patients, raising concerns about its resistance and pathogenesis owing to limited population genetic and genomic information^34^. Recently, the nuclear genomes of two *L. orientalis* strains (PCM2 from the southern province and LSCM4 from the northern province of Thailand) were published, and comparative analysis showed intra-specific genomic similarity^35^. However, the maxicircle and minicircle DNAs of *L. orientalis* remain unknown. Here, we aimed to identify the mitochondrial DNAs of *L. orientalis* strain PCM2 from next-generation and third-generation sequencing data and compare them with previously published maxicircle and minicircle DNAs of other *Leishmania* species. As a result, we presented the first maxicircle sequences and different minicircle classes of *L. orientalis* strain PCM2. These are useful for understanding parasite virulence, the RNA editing process, as well as further investigating the *L. orientalis* population genetics and classification.

## Materials and methods

### Culture of *Leishmania orientalis* strain PCM2

*Leishmania orientalis* strain PCM2 was maintained and provided by the Department of Parasitology, Phramongkutklao College of Medicine, Bangkok, Thailand. The promastigotes were grown at 26 °C in RPMI 1640-modified with 13.3 mM glutamine, 2.5 mM arginine, 0.3 mM cysteine, 1.7 mM glutamate, 62.1 mM proline, 0.6 mM ornithine, 3.8 mM glucose, 2.2 mM fructose, 5.1 mM malate, 2.8 mM α-ketoglutarate, 0.5 mM fumarate, 0.5 mM succinate, 25 mM HEPES, 50 µg/mL gentamicin, 2X MEM vitamins (Gibco, US), and 20% heat-inactivated fetal bovine serum (HIFBS, Gibco, US).

### Genomic DNA preparation

Genomic DNAs of *L. orientalis* were prepared from the late logarithmic phase of the promastigotes. The promastigote pellets (approximately 5 × 10^7^ parasites/mL) were collected and incubated in the lysis buffer (10 mM Tris, 10 mM KCl, 10 mM MgCl_2_, 0.5 M NaCl, 2 mM EDTA, 0.5% SDS, and 10 µL of 10 mg/mL Proteinase K solution) at 60 °C for 1 h until the pellet disappeared. After incubation, the samples were cooled to room temperature before a 1:1 ratio of chloroform-isoamyl alcohol (24:1, v/v) was added and gently vortexed for 30 s. The samples were centrifuged for 15 min at 10,000 rpm, and 300 µL of the aqueous phases were transferred to 1.5 mL tubes. Ten microliters of RNAse (20 mg/mL) were added, and the samples were incubated at room temperature for 3 min. The chloroform-isoamyl alcohol step was repeated to remove RNase. 750 µL absolute ethanol and 100 µL of 7.5 M ammonium acetate were added, and the samples were gently mixed until the solution was homogenous before incubating at -70 °C overnight. The samples were centrifuged for 15 min at 10,000 rpm. After discarding the supernatant, the DNA pellet was washed twice with 70% ethanol. Residual ethanol was carefully removed, and the pellet was air-dried at room temperature. Next, 100–200 µL Tris–EDTA buffer (pH 8.0) was added to the sample tubes and incubated at 37 °C, allowing the DNA pellet to dissolve. All samples were stored at − 70 °C until further use. The DNA concentration was measured using the NanoDrop spectrophotometer (Thermo Fisher Scientific, MA, US), and the genomic integrity was analyzed by 1% agarose gel electrophoresis. The samples with the following criteria: total yield of 100–150 ng/µL, A260/A280 ratio of 1.80 ± 0.05, and A260/A230 ratio of 1.5 ± 0.05, were proceeded through the genome sequencing steps.

### Isolation of maxicircle and minicircle reads from whole genome sequence data

For short-read sequencing, a 101-bp pair-end read library was constructed for whole-genome sequencing using the Illumina HiSeq2000 platform (Illumina, US). For long-read sequencing, a 20-kb PacBio library was constructed using the PacBio standard library preparation methods (Pacific Biosciences, CA) and the SMRTbell Express Template Preparation Kit 1.0. The sequence raw reads were quality-checked by FastQC program version 0.11.9 (http://www.bioinformatics.babraham.ac.uk/projects/fastqc/)^36^ and processed through the filtering and trimming steps with a cut-off value of 30 using BBDuk program in the BBtools pipeline (sourceforge.net/projects/bbmap/). The reads from both platforms were used to generate hybrid assemblies of the circular DNA using the SPAdes assembler version 3.15.3^37,38^, and the parameters were set as –pacbio –plasmid to assemble only the reads that could be constructed into the circular chromosome. Contigs were verified by a BLASTn search against the NCBI database^39^. The maxicircle and minicircle data, including the coding regions, divergent regions, and *12s rRNA* gene from the NCBI database, were downloaded using an in-house Python script. The contigs that shared sequence homology corresponding to the maxicircle of *Leishmania* were selected for further analyses. The rKOMICS package extracted minicircle sequences and polished the contigs from the SPAdes assembly output^40^. This package performed the minicircle sequence cluster (MSC) analysis, which automated the assembly and circularization of mitochondrial genomes. The process of this toolkit was divided into three main steps: (1) assembling the contigs using MEGAHIT before extracting the minicircles using conserved sequences, (2) identifying the start and end of the minicircle for circularization using BLAST, and (3) correcting the direction of the CSB3 motifs for the polishing step. The polishing process generated a minicircle alignment based on the CSB3 sequence (GGGGTTG[G/A]TGTA) to rearrange the wrong sequence orientation in the minicircle contigs. After the assembly and identification, rKOMICS circularized the assembled minicircles using the BLASTn strategy to detect and remove duplicated sequences. Subsequently, rKOMICS polished the circularized minicircle DNAs to ensure the correct orientation of the CSB3 motifs, created minicircle alignments with the CSB1 motif at the starting position, and clustered the contigs based on the minimum percentage of identity using VSEARCH program^41^.

### Annotation and visualization of the maxicircle and minicircle sequences

The extracted maxicircle contigs were annotated manually using the kDNA of *Leishmania enriettii* strain LEM3045 (GenBank accession number: BK010880.1) as a reference. The maxicircle genome was visualized and explored using SnapGene^®^ software version 6.1.1 (https://www.snapgene.com/). The circularized and extended minicircle genomes were annotated using the BLASTn program^39^. Gene annotation and orientation of the maxicircle sequence were compared between different *Leishmania* species and visualized using the progressiveMauve alignment program^42^. The *L. orientalis* maxicircle scaffold sequences were validated by realigning with long-read sequence data using the minimap2 program version 2.14^43,44^ before polishing with the short-read data using the Pilon program version 1.24^45^. Quality and precision of the alignment results were assessed based on the read depth coverage using the samtools program version 1.9^46^ and visualized by the SeqMonk program version 1.48.1 (https://www.bioinformatics.babraham.ac.uk/projects/seqmonk/).

### Phylogenetic analysis of the whole sequences and specific regions of the *L. orientalis* maxicircle and minicircle DNAs

Phylogenetic trees were constructed using the whole sequence, coding regions, divergent regions, and *12s rRNA* gene of the maxicircle DNA and conserved sequence blocks (CSBs) regions from minicircles of *L. orientalis* strain PCM2 compared with those of other *Leishmania* species. The MAFFT program version 7^47^ on the Unipro UGENE software suit version 44.0^48^ was used to align the maxicircle DNAs using the DNA gap open penalty of 1.53, the DNA gap extension penalty of 0.123, and the omitted manual refinements. The pairwise comparison of the maxicircle sequences was conducted using the EMBOSS Water program with the following parameters: EDNAFULL for matrix, the gap penalty of 10.0, and the extended penalty of 0.5^49^. Phylogenetic relationships were inferred using the Randomized Axelerated Maximum Likelihood (RaxML) program version 8^50^ with the bootstrap values of 10,000 replicates for the complete maxicircle, coding regions, divergent regions, and the *12s rRNA* gene as well as those of minicircles. For the phylogenetic analysis, the FastTree program version 2.1^51^ and Interactive Tree Of Life (iTOL) version 5^52^ were used to construct a phylogenetic tree, and the tree labels were adjusted using the MEGA 11 program^53^.

## Results

**Figure 1:**
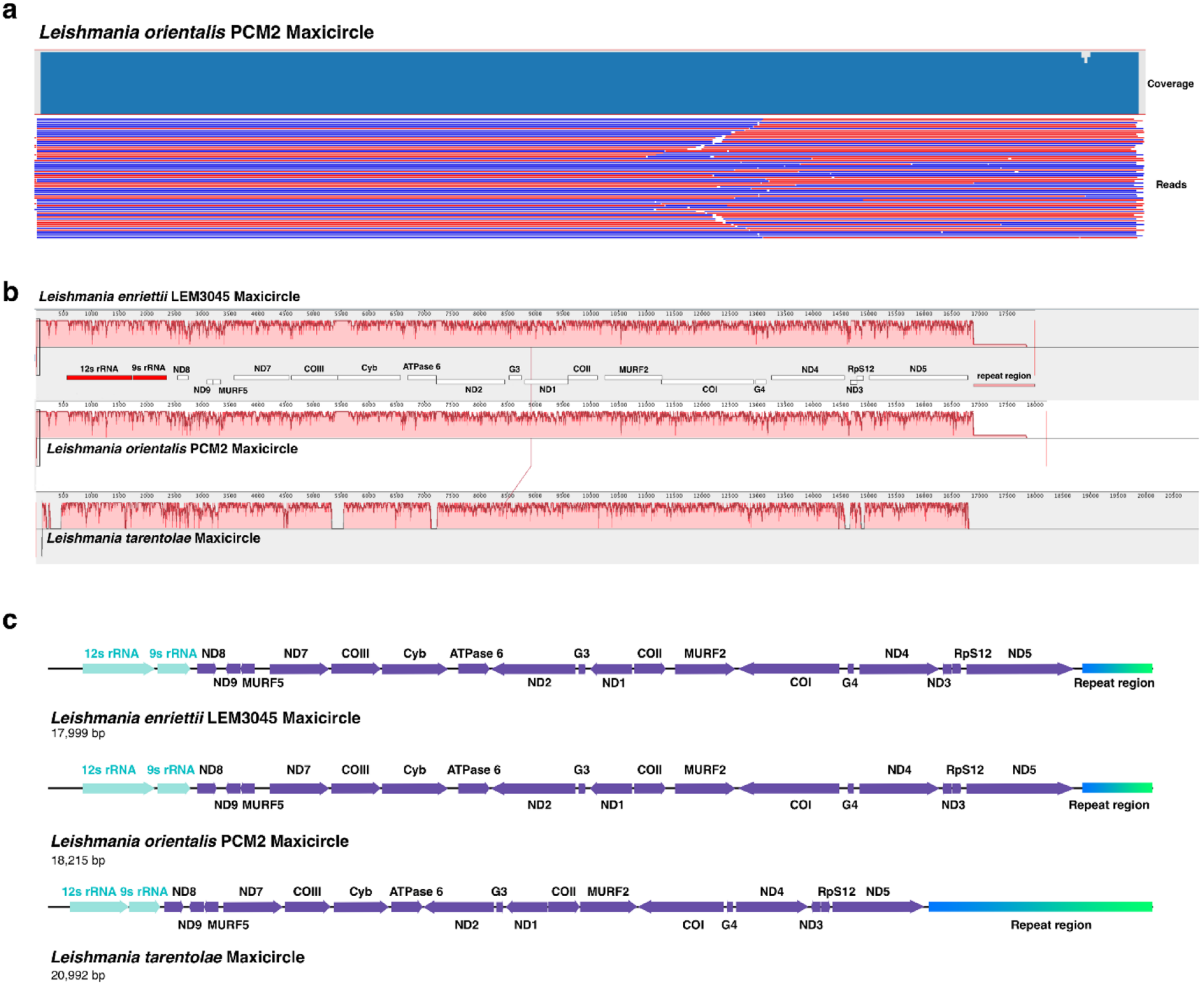
Validation and comparative analysis of the *L. orientalis* maxicircle DNAs with other *Leishmania* species. The long-read data were re-mapped to the assembled maxicircle DNA of *L. orientalis* strain PCM2 to validate the assembly confidence (**a**). The percentage of coverage is shown in the blue bar graph. The maxicircles of *L. enriettii* strain LEM3045, *L. orientalis* strain PCM2, and *L. tarentolae* were structurally compared using the progressiveMauve alignment program (**b**). Distinct coloured blocks denoted regions of RNA genes (red), protein-coding genes (white), and the divergent region (pink). The red bar graph represents the percentage of similarity. The maxicircle gene annotation and arrangement of these three *Leishmania* species included the protein-coding genes (purple), rRNA genes (light green), and divergent regions (blue–green) (**c**). Arrows indicate gene transcription directions.

This study reports the kinetoplastid genome assembly of *L. orientalis* strain PCM2 from an integrative analysis of short-read and long-read whole-genome sequencing data. The maxicircle scaffolds of *L. orientalis* strain PCM2 were validated by the alignment of the PacBio sequencing reads back to the scaffold, and a high level of read coverage (99.52%) was achieved throughout the scaffold length. This output highlighted the robustness and fidelity of the mapping procedure, indicating a comprehensive selection of the maxicircle sequences mixed within the whole genomic read data (Fig. 1a). The de novo assembly of the maxicircle genome using SPAdes resulted in a circular DNA contig of 18,215 base pairs, similar to the complete kinetoplast of *L. enriettii* strain LEM3045 (GenBank accession number: BK010880.1) with a length of 17,999 base pairs, e-value of 0.0, 100% coverage, and 99.92% identity. The *L. enriettii* LEM3045 kinetoplast sequence was shorter because of the different lengths of the divergent region (Fig. 1b). Pairwise alignment of the maxicircle genomes of *L. orientalis* strain PCM2 and *L. enriettii* strain LEM3045 confirmed the high level of relatedness of the subgenus *Mundinia* members, with 98.6% identity, 98.6% similarity, and 1.2% gaps.

To investigate the maxicircle structure, the progressiveMauve alignment program compared the annotated maxicircles of three *Leishmania* species, including *L. tarentolae* (20,992 bp), *L. enriettii* strain LEM3045 (17,999 bp), and *L. orientalis* strain PCM2 (18,215 bp), and showed a more similar genome structure between *L. orientalis* and *L. enriettii* (Fig. 1b). Manual annotation of the *L. orientalis* strain PCM2 maxicircle was conducted by multiple sequence alignment. The annotation revealed the conserved location and orientation of genes and regions closer to the annotated maxicircle of *L. enriettii* strain LEM3045 than those of *L. tarentolae* (Fig. 1c). Similar gene arrangements of the maxicircles of *L. orientalis* strain PCM2, *L. enriettii* strain LEM3045, and *L. tarentolae* were in the following order:* 12s rRNA*, *9 s rRNA*, *ND8*, *ND9*, *MURF5*, *ND7*, *COIII*, *Cyb*, *ATPase 6*, *ND2*, *G3*, *ND1*, *COII*, *MURF2*, *COI*, *G4*, *ND4*, *ND3*, *Rps12*, *ND5*, and DR regions (Fig. 1c). The DR region of *L. tarentolae* was much longer than those of the other two *Leishmania* species.

**Figure 2:**
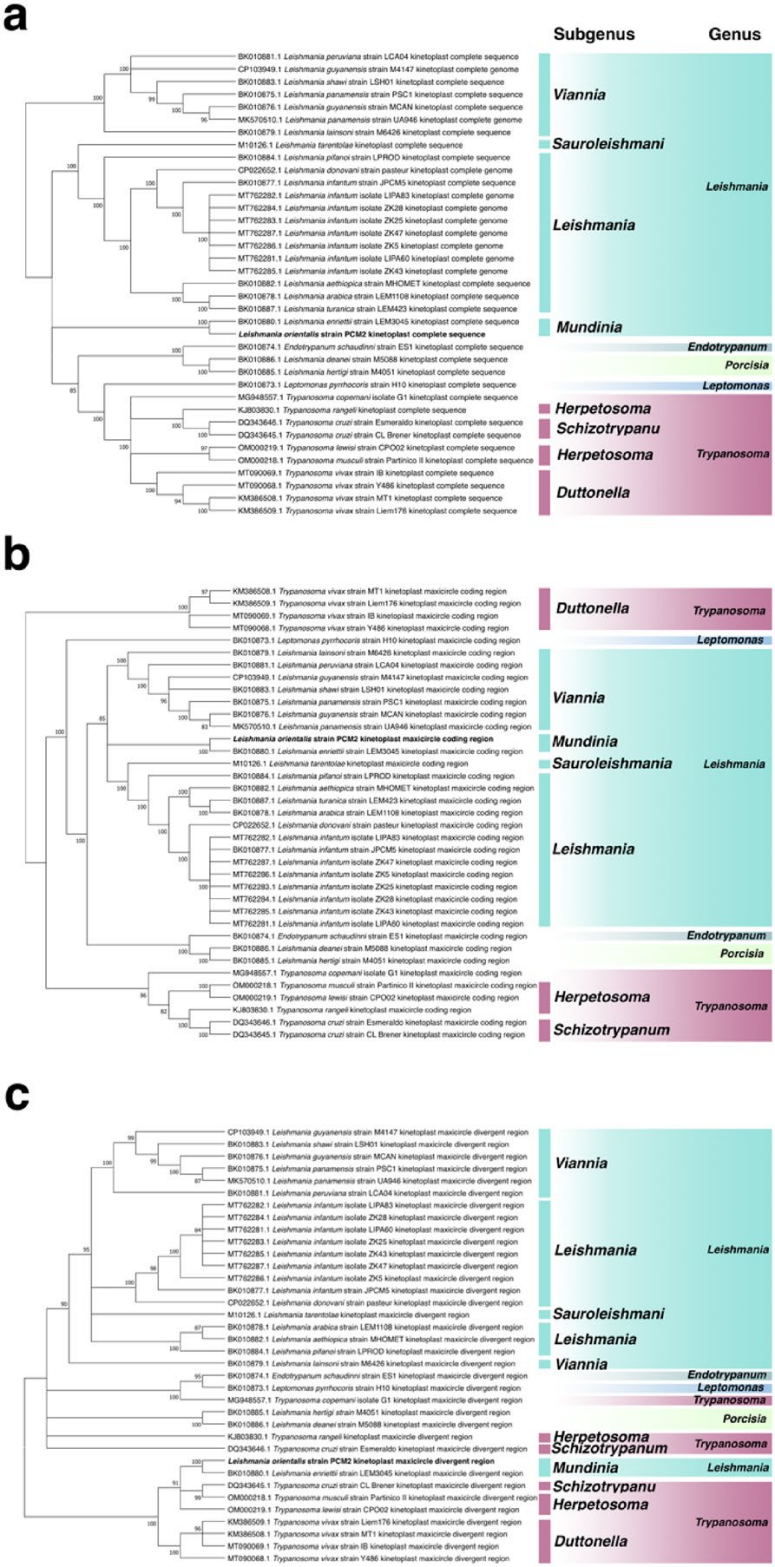
Phylogenetic tree of 22 maxicircles of *Leishmania* and outgroups (*Endotrypanum schaudinni*, *Trypanosoma*, and *Leptomonas pyrrhociris*) obtained from the NCBI database compared with those of *L. orientalis* strain PCM2 and *L. enriettii* strain LEM3045. The whole sequence of maxicircles (**a**), coding regions (**b**), and divergent region (**c**) were analyzed using the Randomized Axelerated Maximum Likelihood method with the GTR + I + G4, GTR + I + G4, and TPM3uf + I + G4 models, respectively. Bootstrap values were derived from 10,000 repetitions. The numbers lower than 80 were not presented. Highlighted colours showed different genera and sub-genera of *Leishmania*.

To investigate the evolution and similarity of *Leishmania* maxicircles, the complete maxicircle sequence of *L. orientalis* PCM2 was compared with that of other *Leishmania* species. The results showed that the maxicircle could be used to separate different subgenera of *Leishmania* (Fig. 2a). The close relationship between the maxicircles of *L. orientalis* strain PCM2 and *L. enriettii* strain LEM3045 was supported by the phylogenetic results of the coding and divergent regions (Figs. 2b and 2c). Besides the highly conserved structure of trypanosomatid maxicircles^54,55^, the DR region, which consists of repeating sequences and displays variations across species^56^ was also selected and examined. The phylogenetic tree of the *Leishmania* DR regions showed the separation of the subgenus *Mundinia* from other *Leishmania* subgenera, as well as the split of the DR regions of the subgenera *Leishmania* and *Viannia* into two clusters, similar to the multiple-group separation of *Trypanosoma* (Fig. 2c). Therefore, the DR region may still be used to distinguish the subgenus *Mundinia* from other *Leishmania* subgenera (Fig. 2c).

**Figure 3:**
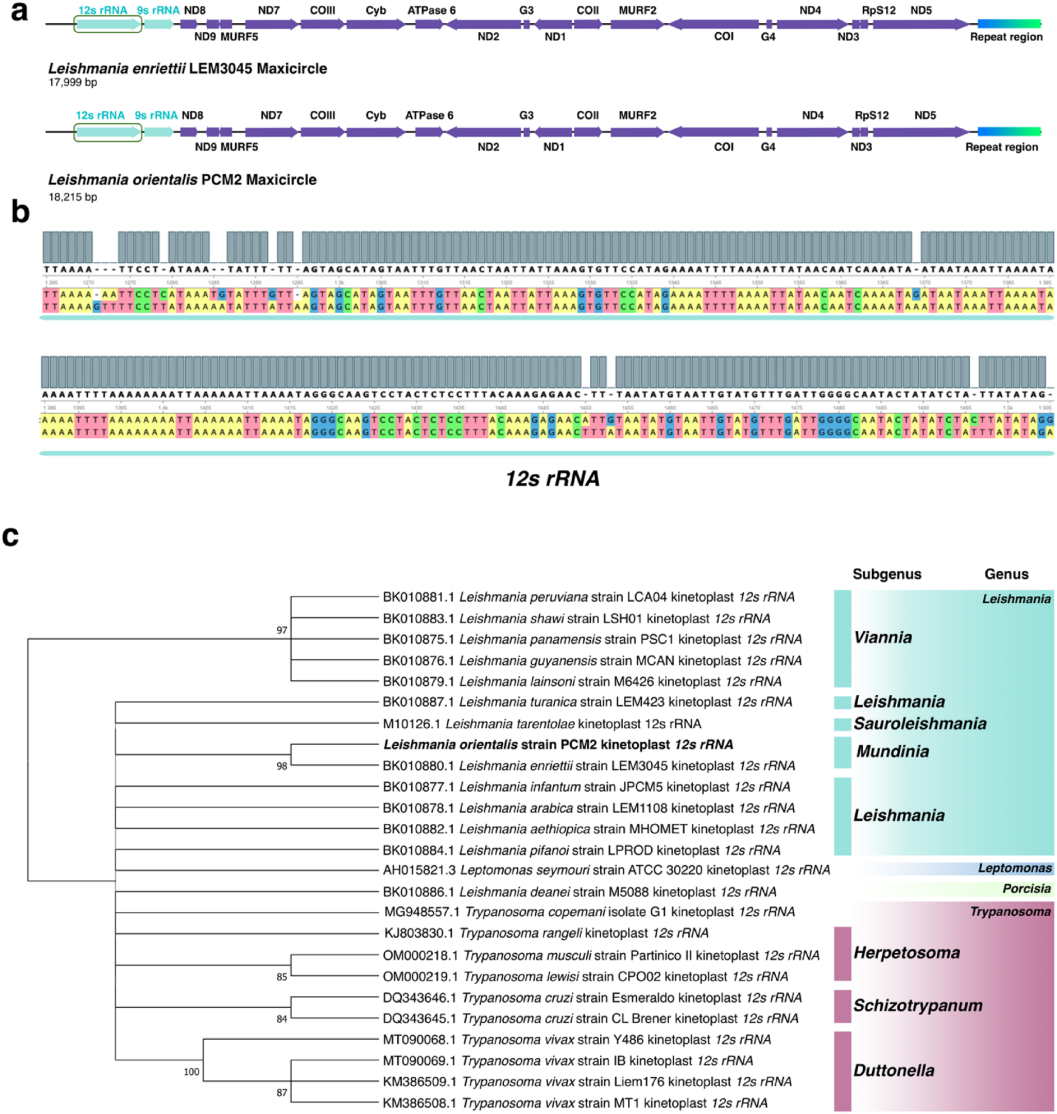
Comparative analysis of *12s rRNA* gene on the *Leishmania* maxicircle DNAs. (**a**) Position of the *12s rRNA* gene (marked by the green box) on the maxicircle DNA of *L. enriettii* strain LEM3045 and *L. orientalis* strain PCM2, and their nucleotide sequence variations were represented in (**b**). The ‘–’ in the row of coloured nucleotides indicated the indel position. (**c**) Phylogenetic tree of *12s rRNA* gene on the maxicircles of 14 *Leishmania* species with those of *Trypanosoma* and *Leptomonas seymouri* as outgroups obtained from the NCBI database. The Randomized Axelerated Maximum Likelihood method and the TrN + G4 model were used for the phylogenetic analysis. Bootstrap values were derived from 10,000 repetitions. The numbers lower than 80 were not presented. Highlighted colours showed different sub-genera of *Leishmania*.

A 0.02% difference between *L. orientalis* strain PCM2 and *L. enriettii* strain LEM3045 was observed not only in the DR region but also in the *12s rRNA* gene region between positions 1270 and 1505 (Figs. 3a and 3b). Eleven SNPs and two deletions were detected when comparing the *12s rRNA* gene of these two *Leishmania* species. A phylogenetic tree of the *12s rRNA* genes from different *Leishmania* species was constructed. It showed that the variants within this gene could be used to distinguish *L. orientalis* strain PCM2 from *L. enriettii* strain LEM3045 and to discriminate subgenus *Mundinia* from other subgenera and outgroups (Fig. 3c).

**Figure 4:**
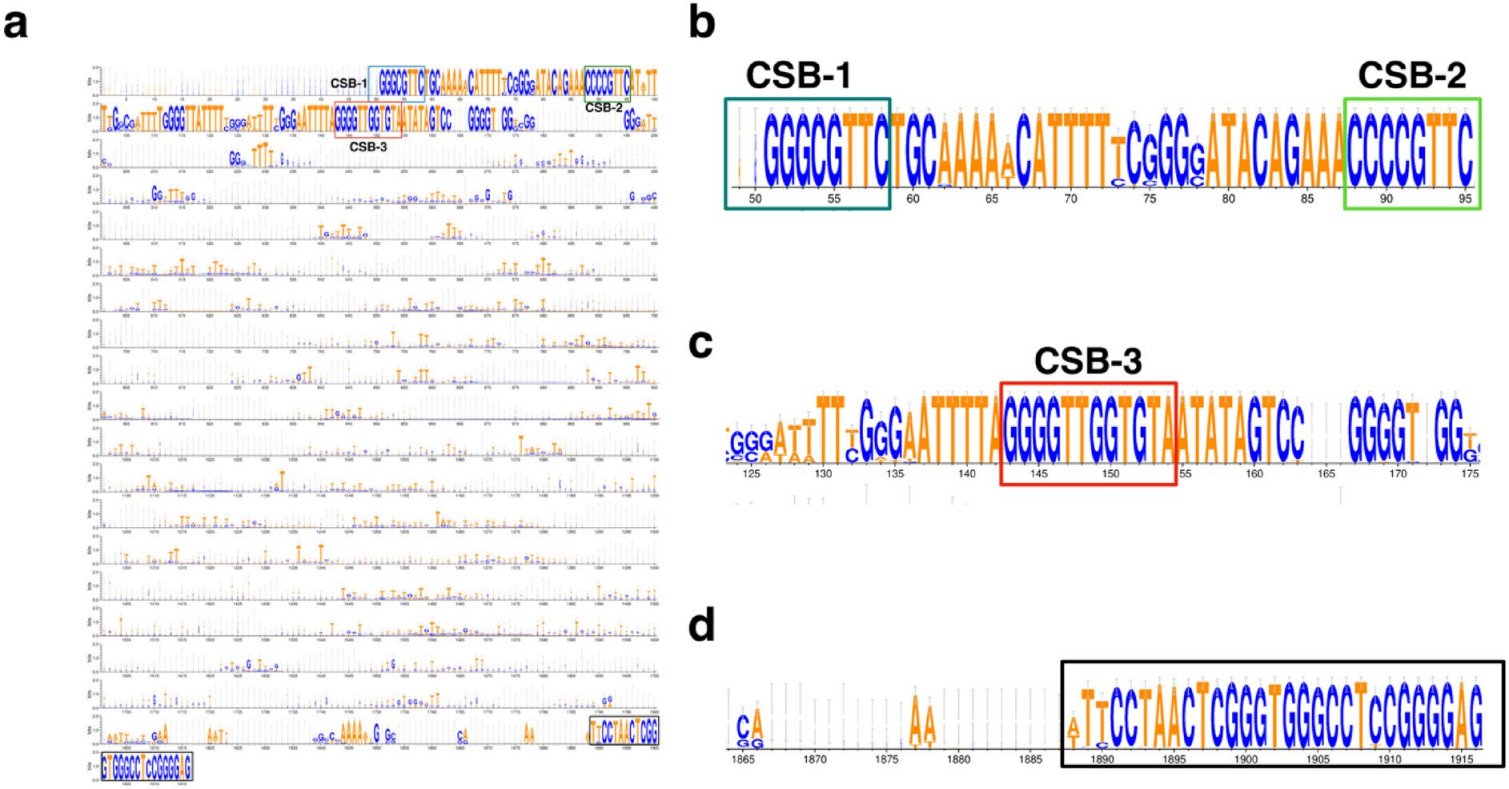
Nucleotide sequence alignment of minicircle DNAs found in *Leishmania orientalis* strain PCM2. The alphabet plot was used to visualize the full-length sequence alignment of 105 minicircle classes by the MAFFT and Weblogo3 programs (**a**). The first conserved region included three conserved motifs: CSB-1, CSB-2, and CSB-3 (**b** and **c**), and the second conserved region was unique to the minicircle of *L. orientalis* strain PCM2 (**d**).

One hundred and five minicircle classes of *L. orientalis* strain PCM2 were identified and compared. Three conserved sequence blocks (CSBs)^57^ were detected in the conserved regions of these minicircles, representing the minicircle signature (Fig. 4a). The identification of CSB-1 (AgGGGCGTTC), CSB-2 (cCCCGTNC), and CSB-3 (GGGGTTGGTGTA) confirmed the accuracy of the minicircle contigs of *L. orientalis* strain PCM2 (Figs. 4b and 4c). Another conserved region at position 1888–1915 was identified by multiple sequence comparisons between these *L. orientalis* minicircles, indicating the within-species similarity of these minicircle DNAs (Fig. 4d).

**Figure 5:**
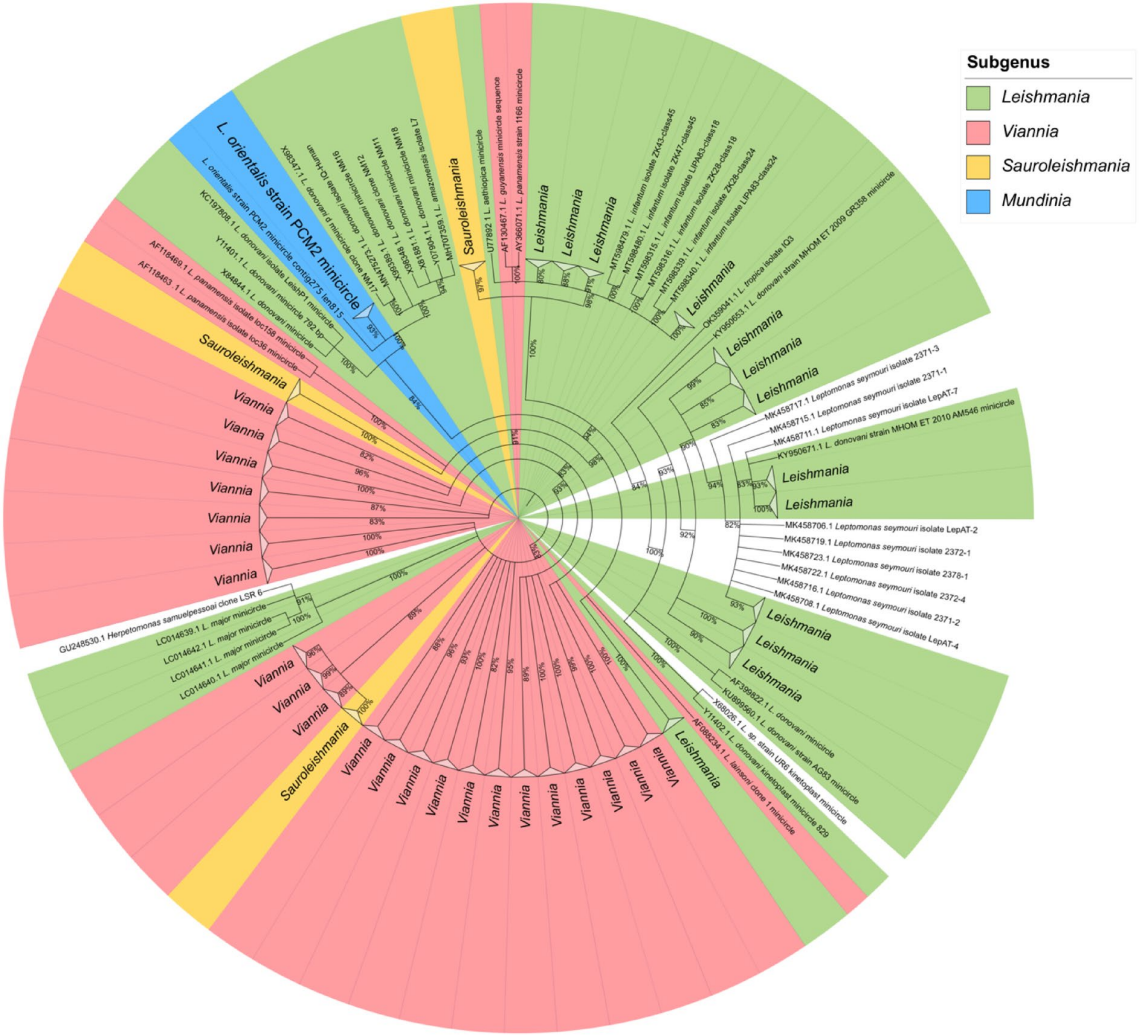
The Randomized Axelerated Maximum Likelihood (RaxML) phylogenetic tree of 105 classes of minicircle DNAs of *Leishmania orientalis* strain PCM2 and 2111 sequences from other *Leishmania* species. The minicircles of *Leptomonas seymouri* and *Herpetomonas samuelpessoai* were defined as outgroups. The local bootstrap support values showed at the branch point, and some branches of the *L. orientalis* strain PCM2 minicircles were collapsed for clear visualization. The concealed clades were represented as triangles at the end of branches. Sequences with values less than 80 were not displayed. Different sub-genera of *Leishmania* were highlighted using distinct colours.

Comparison of 105 minicircle sequences of *L. orientalis* strain PCM2 with 2111 minicircle DNAs of other *Leishmania* species from the NCBI database showed that most of the *L. orientalis* strain PCM2 minicircles were monophyletically arranged within the same clade as some of the *L. donovani* minicircles. In contrast, subgenera Leishmania, Viannia, and Sauroleishmania minicircles were split into multiple groups, indicating the genetic diversification of these minicircles within the same *Leishmania* species (Fig. 5).

## Discussion

This study successfully identified the mitochondrial genome, including a maxicircle and several minicircles, of *L. orientalis* strain PCM2 to complement its nuclear genome, which was previously published^35^. A new molecular-integrated taxonomic scheme for *Leishmania* suggested dividing *Leishmania* species into two major evolutionary lineages, *Euleishmania* and *Paraleishmania*^58^. Since 1999, *L. martiniquensis* has been discovered in many CL and VL cases in Thailand and is genetically linked to *L. enriettii*^59,60^. The subgenus *Mundinia* was established in 2018 and included several *Leishmania* species responsible for human and animal illnesses, *L.* (*Mundinia*) *enriettii*, *L.* (*Mundinia*) *martiniquensis*, formerly assigned to the subgenus *Leishmania*, and *L.* (*Mundinia*) *orientalis*, formerly named *L. siamensis*, a recently discovered species responsible for CL in Thailand^59,61^. Our previous study illustrated the genomic differences between the two strains of *L. orientalis* in Thailand^35^. The findings raised several questions regarding the genetics and population diversity of *L. orientalis* in Thailand and neighbouring countries. The hybrid assembly of the short- and long-read sequence data allowed the successful discovery of a complete maxicircle strand, which was similar to that of *L. enriettii* in terms of sequence and structural arrangement (Fig. 1), and unique classes of 105 minicircles that shared conserved motifs with those of other *Leishmania* species (Figs. 4 and 5).

As the maxicircle of *Leishmania* was previously reported to be useful for inferring phylogenetic relationships^61^, different formats of maxicircle DNAs, i.e. complete sequence, coding regions, divergent regions, and specific genes (*12s rRNA*) were explored in this study. Despite the high similarity (99.98%) between the maxicircles of *L. orientalis* strain PCM2 and *L. enriettii* strain LEM3045, subtle differences were exclusively observed in the *12s rRNA* gene and the highly variable or divergent regions. Phylogenetic analysis of these sequences consistently confirmed the close genetic relationship between *L. orientalis* and *L. enriettii* within the subgenus *Mundinia,* which was nicely separated from other subgenera (Figs. 2 and 3), supporting the proposed usage of the maxicircle for evolutionary examination within the population or the same subgenus of *Leishmania*.

The divergent region is one of the problematic parts of maxicircle assembly because of the repetitive sequences in this region, and its function remains to be investigated^8,62,63^, unlike the coding region (CR), which contains fewer repeats. This study employed a hybrid assembly approach that combined short- and long-read sequencing platforms to enhance the coverage of the divergent region with minimized errors compared to short-read data alone, which was not long enough to cover the repeat area. Interestingly, the observed variation within this divergent region was also closely shared among members of the subgenus *Mundinia* (Fig. 2c), consistent with a previous study on the divergent region in Trypanosomatidae, which could be used to distinguish between different *Leishmania* species^56^. This finding was supported by a study by Flegontov et al., who identified conserved repeat patterns in the divergent region of six *Leishmania* species, including *L. tarentolae*, *L. amazonensis*, *L. mexicana*, *L. major*, *L. turanica*, and *L. chagasi*, using PCR amplification and Sanger sequencing^7,23^. Therefore, the uniqueness of the divergent region of *L. orientalis* maxicircle could benefit further investigation of *L. orientalis* population genetics and diversity in this Southeast Asian area.

In contrast, subtle variations were detected only in the coding region of the *12s rRNA* gene of the maxicircles of *L. orientalis* and *L. enriettii* (Fig. 3). This gene is a mitoribosomal RNA gene that plays a role in the translation process and is recognized as a highly conserved gene in the Trypanosomatidae family^56^. The 12s rRNA variation was also observed in other *Leishmania* and trypanosome species, suggesting that this gene variation was perhaps the first event that occurred during the divergence of these two members in the subgenus *Mundinia*. The trypanosomal mitochondrial rRNAs, including 12S and 9S rRNAs, are substantially smaller than the eubacterial 23S and 16S rRNAs and also smaller than the mammalian mitochondrial 16S and 12S rRNAs due to the missing stem-loop structure in the secondary structure^64,65^. This variation may affect rRNA stability, secondary structure, and translation process^66^. The variable positions of the *12s rRNA* gene were thus helpful for distinguishing between *L. orientalis* and *L. enriettii* and for further improvement of species-specific diagnostic methods.

The second part of the *Leishmania* kDNA network contains several thousands of minicircles ranging from 0.5 to 2 kbp, which transcribe guide RNAs required for the RNA editing process of maxicircle gene transcripts^3^. These guide RNAs bind to mitochondrial mRNA and edit the targeted mRNA sequences^5,67,68^. In *Trypanosoma brucei*, transcript editing accounts for over half of mature mRNAs^69^. According to the large number of minicircles (approximately 10,000 molecules classified into different classes) per cell, these minicircle DNAs were previously used as another suitable target for PCR-based *Leishmania* diagnosis^70–72^. The present and previous evolutionary studies have consistently distinguished the Old World (*L. donovani*, *L. infantum*, *L. chagasi*, *L. tropica*, *L. major*, and *L. aethiopica*) and New World (*L. mexicana*, *L. amazonensis*, *L. venezuelensis*, *L. braziliensis*, *L. guyanensis*, *L. panamensis*, and *L. peruviana*) *Leishmania* species using minicircle DNA analysis^73–75^.

Interestingly, in the present study, different classes of minicircles were detected in *L. orientalis* strain PCM2, which could be classified in the same clade and were closely related to *L. donovani* minicircles (Fig. 5). This study also identified an *L. orientalis*-specific conserved region in all samples between positions 1888 and 1916 (A/TTCCCCTAACTCGGGTGGGCCTCCGGGGGAG) of their minicircle DNAs, which was not related to any known minicircle DNAs in the NCBI nucleotide database (Fig. 4d). However, minicircle data of the subgenus *Mundinia* remain limited, and the inter- and intra-specific relationships within this genus could be further expanded using minicircle DNAs^76^. Considering the kDNA function, maxicircle DNAs function as mtDNA of other organisms, while minicircle DNAs play crucial roles in RNA editing^77^. The high genetic heterogeneity of these minicircles was previously reported and showed that different minicircular sequences could drive similar editing functions, but perhaps on different groups of genes^78–80^. The minicircular variation could also be divided into more than 200 classes within the same species, suggesting unexplained adaptable and pathogenic mechanisms to be further investigated^81^ as well as possible different functions of these different guide RNA genes^82^. Therefore, the unique sequences observed in the maxicircle and minicircle DNAs of *L. orientalis* strain PCM2 may be useful for a better specific diagnosis and identification of *L. orientalis* from more samples in the future.

## Conclusion

This study was the first report to identify the mitochondrial genome or kDNA of *L. orientalis* strain PCM2, comprising a maxicircle and 105 minicircle classes, using hybrid whole-genome sequencing data. The maxicircle displayed a conserved genomic structure and gene arrangement in closely related *Leishmania* species. Variations in maxicircle and minicircle DNAs would benefit species identification and population genetic studies. Classes and sequence variations of minicircle DNAs could affect parasite adaptation and pathogenesis via RNA editing. Further investigation of the maxicircle and minicircle of more *L. orientalis* isolates would allow monitoring of this parasite for better medical treatment and healthcare.

## Data Availability

The data supporting this study's findings are submitted to the NCBI GenBank database, accession numbers OP159070-OP159175.
